# Highly Correlated Multimorbidity in Alzheimer’s Disease: An EHR Analysis

**DOI:** 10.1093/geroni/igaf122.2855

**Published:** 2025-12-31

**Authors:** Nai-Ching Chi, Kathleen Buckwalter, Pui Ying Yew, Nanle Gusen, Hua-Chin Chi, Chih-Lin Chi, Scott Larson

**Affiliations:** University of Iowa, Iowa City, Iowa, United States; University of Iowa, Iowa City, Iowa, United States; University of Minnesota, Minneapolis, Minnesota, United States; University of Iowa, Iowa City, Iowa, United States; Emory University, Atlanta, Georgia, United States; University of Minnesota, Minneapolis, Minnesota, United States; University of Iowa, Iowa City, Iowa, United States

## Abstract

Most multimorbidity studies focus on the prevalence of individual chronic conditions, neglecting the crucial aspect of correlated disease combinations. This study addressed this gap by investigating highly correlated multimorbidity patterns in 2,629 Alzheimer’s Disease (AD) patients using de-identified electronic health records from the University of Iowa Hospitals and Clinics. Diagnoses, coded to the first three levels of ICD-10, were analyzed using the Apriori algorithm to identify significant co-occurring chronic conditions (dyads, triads, and tetrads). Leverage metrics quantified the strength of these correlations. The hypertension-lipoprotein metabolism disorders dyad (I10-E78) was most prominent, occurring 7.8% more frequently than expected (21.4% total). Other significant dyads included depression-anxiety (F33-F41; 4.5% leverage), dyslipidemia-ischemic heart disease (E78-I25; 3.9% leverage), hypertension-ischemic heart disease (I10-I25; 3.7% leverage), and type 2 diabetes-dyslipidemia (E11-E78; 3% leverage). Key triads included hypertension-dyslipidemia-depression (I10-E78-F33; 4.5% leverage), hypertension-dyslipidemia-ischemic heart disease (I10-E78-I25; 4.5% leverage), hypertension-dyslipidemia-type 2 diabetes (I10-E78-E11; 4.2% leverage), hypertension-dyslipidemia-gastroesophageal reflux (I10-E78-K21; 3.9% leverage), and hypertension-dyslipidemia-hypothyroidism (I10-E78-E03; 3.4% leverage). The hypertension-dyslipidemia-depression-anxiety tetrad (I10-E78-F33-F41; 2.6% leverage) exhibited the strongest correlation. Additional significant tetrads revealed complex interactions involving gastroesophageal reflux, sleep disorders, ischemic heart disease, and hypothyroidism. These findings demonstrate a significant clustering of cardiovascular, metabolic, and psychiatric comorbidities in AD. Identifying these correlated multimorbidity clusters will enable the development of targeted, integrated management strategies, inform evidence-based clinical guidelines, and facilitate focused clinical screening and intervention for this vulnerable population.

